# Multi-parametric MRI in the diagnosis and scoring of gastrointestinal acute graft-versus-host disease

**DOI:** 10.1007/s00330-023-09563-7

**Published:** 2023-04-18

**Authors:** Francesca Maccioni, Ursula La Rocca, Alberto Milanese, Ludovica Busato, Arianna Cleri, Mariangela Lopez, Lucia Manganaro, Carlo De Felice, Cira Di Gioia, Anna Rita Vestri, Carlo Catalano, Anna Paola Iori

**Affiliations:** 1 Department of Radiological Sciences, Pathology and Oncology, Policlinico Umberto I Hospital, Sapienza University of Rome, Viale Regina Elena 324, 00161 Rome, Italy; 2 Department of Translational and Precision Medicine, Policlinico Umberto I Hospital, Sapienza University of Rome, Via Benevento 6, 00161 Rome, RM Italy; 3 Department of Public Health and Infectious Diseases, Sapienza University of Rome, Piazzale Aldo Moro 5, 00185 Rome, Italy

**Keywords:** Graft vs host disease, Magnetic resonance imaging (MRI), Hematopoietic stem cell transplantation, Gastrointestinal tract, Inflammation

## Abstract

**Objectives:**

Acute gastrointestinal graft-versus-host disease (GI-aGVHD) is a severe complication of allogeneic hematopoietic stem cell transplantation (HSCT). Diagnosis relies on clinical, endoscopic, and pathological investigations. Our purpose is to assess the value of magnetic resonance imaging (MRI) in the diagnosis, staging, and prediction of GI-aGVHD-related mortality.

**Methods:**

Twenty-one hematological patients who underwent MRI for clinical suspicion of acute GI-GVHD were retrospectively selected. Three independent radiologists, blinded to the clinical findings, reanalyzed MRI images. The GI tract was evaluated from stomach to rectum by analyzing fifteen MRI signs suggestive of intestinal and peritoneal inflammation. All selected patients underwent colonoscopy with biopsies. Disease severity was determined on the basis of clinical criteria, identifying 4 stages of increasing severity. Disease-related mortality was also assessed.

**Results:**

The diagnosis of GI-aGVHD was histologically confirmed with biopsy in 13 patients (61.9%). Using 6 major signs (diagnostic score), MRI showed 84.6% sensitivity and 100% specificity in identifying GI-aGVHD (AUC = 0.962; 95% confidence interval 0.891–1). The proximal, middle, and distal ileum were the segments most frequently affected by the disease (84.6%). Using all 15 signs of inflammation (severity score), MRI showed 100% sensitivity and 90% specificity for 1-month related mortality. No correlation with the clinical score was found.

**Conclusion:**

MRI has proved to be an effective tool for diagnosing and scoring GI-aGVHD, with a high prognostic value. If larger studies will confirm these results, MRI could partly replace endoscopy, thus becoming the primary diagnostic tool for GI-aGVHD, being more complete, less invasive, and more easily repeatable.

**Key Points:**

*• We have developed a new promising MRI diagnostic score for GI-aGVHD with a sensitivity of 84.6% and specificity of 100%; results are to be confirmed by larger multicentric studies.*

*• This MRI diagnostic score is based on the six MRI signs most frequently associated with GI-aGVHD: small-bowel inflammatory involvement, bowel wall stratification on T2-w images, wall stratification on post-contrast T1-w images, ascites, and edema of retroperitoneal fat and declivous soft tissues.*

*• A broader MRI severity score based on 15 MRI signs showed no correlation with clinical staging but high prognostic value (100% sensitivity, 90% specificity for 1-month related mortality); these results also need to be confirmed by larger studies.*

## Introduction

Allogeneic hematopoietic stem cell transplantation (HSCT) is a potentially curative therapy for malignant and non-malignant hematologic diseases that do not respond to standardized treatments [1–3]. Graft-versus-host disease (GVHD) is a common complication after transplantation and remains a major cause of morbidity and mortality [4–6]. Acute GVHD (aGVHD) classically occurs within 100 days after transplantation in 30–50% of patients; approximately 14–36% of patients develop severe aGVHD [7–11]. It commonly affects the skin, gastrointestinal (GI) tract, and/or liver. The intestinal tract is affected in approximately half of the cases. Symptoms are nonspecific, including diarrhea, abdominal pain, and paralytic ileus [12, 13]; there is a large clinical overlap with other acute GI disorders following HSCT, such as cytomegalovirus enterocolitis, pseudomembranous colitis, and neutropenic colitis [4, 14].

The diagnosis of GI-aGVHD is challenging but essential for effective management of the disease. Endoscopy of the upper and lower gastrointestinal tract with multiple biopsies is the gold standard in the differential diagnosis between GVHD and other gastrointestinal infectious diseases, although often contraindicated due to the severe clinical status of these patients [15]. The severity of the disease is usually clinically assessed according to the Glucksberg criteria [16], which are based only on the volume of diarrhea and gastrointestinal symptoms (Table 1).

**Table 1:** Glucksberg staging system. Clinical staging system of gastrointestinal acute GVHD, according to the Glucksberg criteria. Severity depends on the volume of diarrhea and gastrointestinal symptoms

Stage	Quantity of diarrhea and gastrointestinal symptoms
I	500–1000 mL
II	1000–1500 mL
III	> 1500 mL
IV	Severe abdominal pain with and without ileus

In this difficult clinical setting, imaging can play an important role. Most published studies are based on CT, ultrasound, and more recently PET [17–25], whereas few data exist on magnetic resonance imaging (MRI) [26–28]. However, MRI is nowadays considered an effective tool for the diagnosis of inflammatory bowel diseases [29, 30], and is regarded as the gold standard for monitoring disease activity in Crohn’s disease [31–34]. Interestingly, recent studies showed a common pathogenesis between IBD and GVHD, both being based on a compromised intestinal barrier function [35, 36]. Similarly, the treatment of aGVHD has recently been enhanced by monoclonal antibodies, analogous to those used in inflammatory bowel disease, with promising results [37–40]. For these reasons, we believe that MRI could also play a relevant role in the diagnostic workup of GI-aGVHD.

The purpose of this study is to evaluate the accuracy of MRI in the diagnosis and staging of the disease severity of GI-aGVHD.

## Materials and methods

This is a retrospective study conducted on patients treated with HSCT, followed at the Hematology Unit of the Department of Translational and Precision Medicine of Policlinico Umberto I Hospital, Sapienza University of Rome. The study protocol was approved by the local ethics committee.

Between January 2015 and January 2021, most of the patients after allogeneic HSCT with clinical suspicion of aGVHD underwent MRI as an alternative to CT for bowel evaluation. Informed consent was obtained from all patients, regarding both diagnostic and therapeutic decisions.

Patients were selected according to the following criteria:

- Age ≥ 18 years
- Clinical suspicion of GI-aGVHD within 100 days after transplantation
- Complete clinical and endoscopic evaluation
- Complete MRI examination of the small and large bowel
- Time interval between MRI and endoscopy < 2 weeks
- Time interval between MRI and evaluation of laboratory parameters < 72 h
- Written informed consent to perform MRI

The exclusion criterion was an incomplete MRI examination.

### Clinical-endoscopic evaluation

The severity of GI-aGVHD was reported according to clinical criteria; patients were stratified into 4 stages and classified according to the widely used clinical staging system based on the Glucksberg criteria [16] (Table 1).

All selected patients underwent endoscopic examination of the upper (esophagogastroduodenoscopy) and lower (rectosigmoid colonoscopy) intestine; biopsy samples were acquired from pathological segments of the upper GI and from each segment of the colon, regardless of inflammatory changes in the mucosa. A histopathological evaluation, still considered the gold standard for the diagnosis of GVHD, was performed in all patients.

### Magnetic resonance imaging

All included examinations were performed using a 1.5-T magnet (Avanto, Siemens, Erlangen, Germany) with a 16-channel phased-array coil, including the following technical aspects.

Gadolinium-based contrast agent (Claricyclic-Clariscan®, GE Healthcare) was intravenously administered at a dose of 0.1 mmol/kg or 0.2 mL/kg. Oral contrast medium (polyethylene glycol solution) was administered in varying amounts depending on the patient’s weight, age, and patient compliance; the average dose was 1.5 l, administered 45 min before the start of the examination, in order to obtain adequate distension of the small intestine up to the last ileal loop (MR enterography) according to standard recommendations [31]. In severely compromised patients, however, no oral contrast could be administered, or only smaller amounts.

- The MRI protocol included the following sequences: axial and coronal T2-weighted half Fourier acquisition single-shot turbo spin echo (HASTE) sequence, with and without fat suppression (TR 1000 ms, TE 83 ms, flip angle 150°, and 5-mm slice thickness); these sequences are not affected by motion-peristaltic artifacts and may be acquired with free-breathing scans.
- Axial and coronal true fast imaging steady-state free precession (TrueFISP) (TR 4 ms, TE 1 ms, flip angle 60°, and 4-mm slice thickness).
- Axial single-shot fat-suppressed echo-planar diffusion-weighted sequence (TR 8000 ms, TE 70 ms, and 5-mm slice thickness) with a *b* value of 0, 500, and 1000 s/mm^2^.
- Axial contrast-enhanced T1-weighted volumetric interpolated breath-hold examination (VIBE) with fat suppression (TR 6 ms, TE 3 ms, flip angle 10°, and 3–4-mm thickness), starting about 20 s after gadolinium administration with an arterial phase, followed by a venous acquisition and a late coronal acquisition. Before contrast injection, a 20-mg dose of hyoscine butylbromide (Buscopan®, Boehringer Ingelheim) was intravenously administered to reduce bowel motion artifacts.

### Image analysis

Images of selected studies/patients were retrospectively analyzed by three independent readers, blinded to clinical and radiological data. The first was the referring senior radiologist (F.M.); the second (L.M.), a senior radiologist; the third (M.L.), a 4-year resident. The inter-observer agreement was calculated; in case of disagreement, the final decision was achieved by consensus.

For this analysis, the GI tract was arbitrarily divided into 12 segments: stomach; duodenum; jejunum; proximal, middle, and distal ileum; cecum; ascending, transverse, and descending colon; sigma; and rectum. Five segments pertained to the small bowel and 5 to the colon, plus stomach and rectum.

Fifteen different MRI parameters were considered to be suggestive of intestinal and/or peritoneal inflammation and preselected to assess the severity of the GI-aGVHD on the basis of previous studies on GVHD [17–28], previous studies on IBD [30–33], and our team experience. Once one or more pathological intestinal segments were identified, their location was reported on a database, their length measured, and each single parameter assessed.

These parameters are listed in Table 2. Each of the preselected 15 MRI parameters was quantified (qualitatively or quantitatively), according to a 0–1, 0–2, or 0–3 point-based system, in order to obtain a final *MRI severity score* ranging between 0 and 27 points, inclusive of all parameters. This score was correlated to the clinical score (Glucksberg score) and to 1-month mortality.

**Table 2:** MRI parameters used for the MRI severity score

Parameters	Points	No. of GI-aGVHD patients	No. of non-GI-aGVHD patients	*p* value
*1. Number of involved segments*	0: no involved segments	0/13 (0%)	0/8	0.163
	1: 1–3 segments	2/13 (15.4%)	2/8 (25%)	
	2: 4–6 segments	4/13 (30.1%)	5/8 (62.5%)	
	3: > 6	7/13 (53.8%)	1/8 (12.5%)	
*2. Maximum wall thickness*	0: < 3 mm	2/13 (15.4%)	0/8	0.146
	1: 3–6 mm (mild)	8/13 (61.5%)	3/8 (37.5%)	
	2: 6–10 mm (moderate)	3/13 (23.1%)	3/8 (37.5%)	
	3: > 10 mm (severe)	0/13 (0%)	2/8 (25%)	
3. Wall T2-weighted signal* [assessed on T2-weighted fat-suppressed images]	0: absent/low	1/13 (7.7%)	2/8 (25%)	0.516
	1: moderate	6/13 (46.1%)	2/8 (25%)	
	2: high	6/13 (46.1%)	4/8 (50%)	
4. T2-weighted stratification** [assessed on T2-weighted fat-suppressed images]	0: absent/low	2/13 (15.4%)	5/8 (62.5%)	**0.014**
	1: moderate	3/13 (23.1%)	3/8 (37.5%)	
	2: high or marked	8/13 (61.5%)	0/8	
5. T2-weighted edema of the mesenteric adipose tissue*** [assessed on T2-weighted fat-suppressed images]	0: absent/low	1/13 (7.7%)	6/8 (75%)	**0.006**
	1: moderate	5/13 (38.5%)	1/8 (12.5%)	
	2: high	7/13 (53.8%)	1/8 (12.5%)	
*6. Post-contrast T1-weighted wall enhancement* [assessed in the arterial phase]	0: absent	1/12 (8.3%)	0/8	**0.012**
	1: moderate-obvious (visible, lower than Gd enhancement of the renal medulla)	4/12 (33.3%)	8/8 (100%)	
	2: high (equal to or higher than Gd enhancement of the renal medulla)	7/12 (58.3%)	0/8	
*7. Post-contrast parietal stratification* [better assessed in the arterial phase]	0: absent	3/12 (25%)	8/8 (100%)	**0.004**
	1: present	9/12 (75%)	0/8	
*8. Continuous intestinal involvement (of the small and/or large bowel)*	0: absent	1/13 (7.7%)	1/8 (12.5%)	1
	1: present	12/13 (92.3%)	7/8 (87.5%)	
9. Parietal stiffness****	0: absent	2/13 (15.4%)	7/8 (87.5%)	**0.005**
	1: present	11/13(84.6%)	1/8 (12.5%)	
*10. Increased number and/or size of mesenteric lymph nodes*	0: absent or up to 5 lymph nodes, < 12 mm in diameter	9/13 (69.2%)	6/8 (75%)	0.724
	1: > 5 lymph nodes or > 1 lymph node > 12 mm in diameter	3/13 (23.1%)	2/8 (25%)	
	2: > 10 lymph nodes or > 3 lymph nodes > 12 mm in diameter	1/13 (7.7%)	0/8	
*11. Peritoneal effusion*	0: absent	2/13 (15.4%)	5/8 (62.5%)	**0.04**
	1: moderate (evident only or mostly in the Douglas pouch)	6/13 (46.2%)	3/8 (37.5%)	
	2: abundant (evident in most of peritoneal spaces	5/13 (38.5%)	0/8	
*12. Comb sign (engorgement of intestinal vasa recta*)	0: absent	4/13 (30.8%)	5/8 (62.5%)	0.331
	1: present	9/13 (69.2%)	3/8 (37.5%)	
*13. Restricted diffusion of the intestinal wall on DWI*	0: absent	8/13 (61.5%)	4/8 (50%)	1
	1: present	5/13 (38.5%)	4/8 (50%)	
*14. Edema of retroperitoneal adipose tissue (mostly perirenal space)* [assessed on T2-weighted fat-suppressed images]***	0: absent	1/13 (7.7%)	7/8 (87.5%)	**0.001**
	1: moderate	4/13 (30.8%)	0/8	
	2: abundant	8/13 (61.5%)	1/8 (12.5%)	
*15. Edema of declivous tissues (muscle and subcutaneous tissues mostly in declivous abdominal and pelvic regions)* [assessed on T2-weighted fat-suppressed images]***	0: absent	0/13 (0%)	8/8 (100%)	** < 0.001**
	1: moderate	4/13 (30.8%)	0/8	
	2: abundant	9/13 (69.2%)	0/8	

**Note:** Significant *p* values (< 0.05) are reported in “bold”

*T2-weighted wall signal. Point 0/absent: similar to the T2-weighted signal of the psoas muscle. Point 1 or moderate: barely visible but evident, lower than the T2-weighted signal of the renal parenchyma. Point 2 or severe: well evident, equal to or higher than the T2-weighted signal of the renal parenchyma

**T2-weighted wall stratification. Point 0: absent. Point 1 or moderate: barely visible but present, usually associated with grade 1 (mild) wall thickening. Point 2 or severe: well evident, usually associated with grade 2–3 (moderate to severe) wall thickening

***T2-weighted edema of the mesenteric adipose tissue/declivous tissues and muscles/retroperitoneal fat tissue. Point 0: absent. Point 1 or moderate: barely visible but present, lower than the T2-weighted signal of the renal parenchyma. Point 2 or severe: well evident, equal to or higher than the T2-weighted signal of the renal parenchyma

****Intestinal stiffness: intended as a continuous diffuse and circumferential intestinal wall edema (continuous wall thickening) which causes a serpiginous and rigid appearance of the involved bowel, showing smooth rather than sharp angles

Finally, the three readers identified the most significant parameters for the diagnosis of GI-aGVHD on consensus, unblinded to clinical results and in agreement with the statistical evaluation, in order to obtain an effective and shorter MRI score for the diagnosis of GI-aGVHD, resulting in an *MRI diagnostic score.*

We calculated both the diagnostic score (using the most significant parameters) and the prognostic score (using all 15 parameters), selecting the cut-off for diagnosis and prognosis according to Youden’s *J* statistic.

### Statistical analysis

A descriptive analysis was performed for all of the clinical and MRI variables in the GI-aGVHD group: continuous variables were tested for normality using Shapiro–Wilk test and were descriptively summarized using means and standard deviation or median and interquartile range according to the distribution of each variable; categorical variables were reported using counts and percentages.

In both GI-aGVHD and non-GI-aGVHD groups, further analysis was performed for the subset of all the MRI variables in order to assess the presence of statistically significant differences between the aforementioned groups: continuous variables were compared using Student’s *t* test for independent samples or Mann–Whitney *U* test depending on each variable’s normality; categorical variables were tested by means of *χ*2 test. All tests were two-tailed and the level of significance was set at *α* = 0.05.

Inter-rater reliability (IRR) among the 3 independent raters/readers on the MRI parameter estimation was assessed through Fleiss’ kappa in the case of categorical and ordinal variables and through intraclass correlation coefficient for continuous variables. Receiver operating characteristic (ROC) curves, and their confidence intervals, were constructed for the diagnostic and prognostic MRI-based scores proposed here.

The optimal cut-off value was estimated according to Youden’s *J* statistic criterion, and the associated sensitivity and specificity values were reported.

Statistical analyses were conducted using the statistical software R (R Core Team, 2021; R version 4.1.0).

## Results

### Patients

**Fig. 1:**
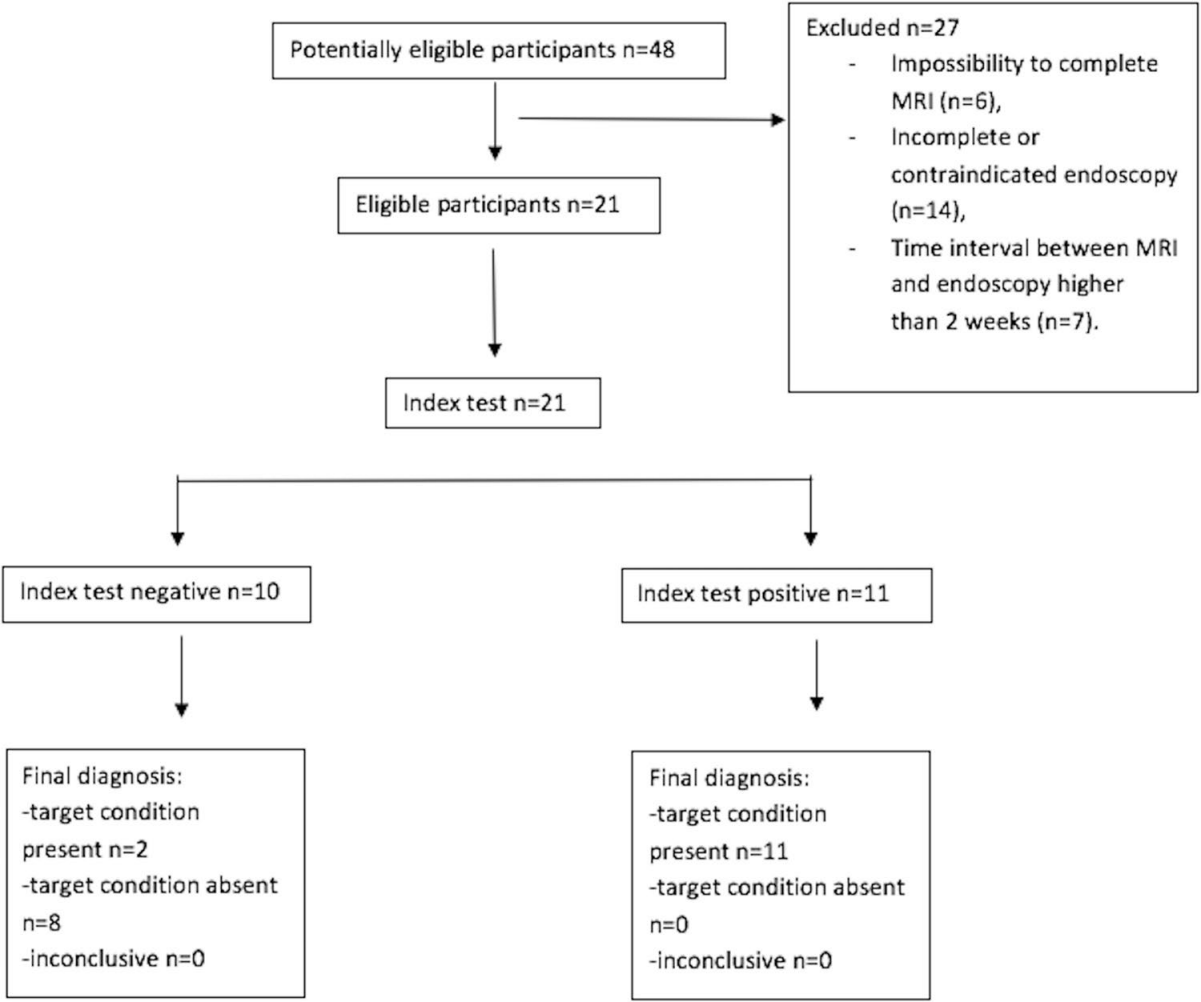
Diagram reporting the flow of participants through the study

A total of 48 patients who had undergone HSCT and with clinical suspicion of GI-aGVHD were referred to MRI; out of these 48 patients, twenty-seven were excluded due to:

- Impossibility to complete MRI for severe clinical conditions or claustrophobia (6/27)
- Incomplete or contraindicated endoscopy (14/27)
- Time interval between MRI and endoscopy longer than 2 weeks (7/27) (Fig. 1)

Twenty-one consecutive patients (11 males, 10 females) were finally included, ranging in age from 18 to 66 years (median age 42 years, interquartile range 36;53.5). The mean time between HSCT and MRI was 68 days (IQR 46;104). The most relevant clinical characteristics of the patients are reported in Table 3.

**Table 3:** Clinical characteristics of the patient population

Characteristic	Value
Number of patients	21 (100%)
Patient age	Median: 42 IQR: 17.5 1st: 36 3rd: 53.5
Sex	
Males	11 (52.4%)
Females	10 (47.6%)
Allogeneic hematopoietic stem cell transplantation (HSCT)	
Matched related donors	11 (52.4%)
Matched unrelated donors	10 (47.6%)
Underlying disease	
Acute myeloid leukemia	8 (38.1%)
Myelofibrosis	4 (19%)
Myelodysplastic syndrome	3 (14.3%)
Acute lymphoid leukemia	3 (14.3%)
Chronic lymphoid leukemia	1 (4.8%)
Chronic myeloid leukemia	1 (4.8%)
Post-LLC Richter syndrome	1 (4.8%)
Hematopoietic stem cell origin	
Bone marrow	9 (42.9%)
Peripheral blood	12 (57.1%)
GVHD location (on 13 patients)	
Skin	11 (84.6%)
Mucosae	2 (15.4%)
GI	13 (100%)
Liver	6 (46.2%)
Lung	0 (0%)
Muscular	0 (0%)
Other	0 (0%)
GI-GVHD clinical grading (on 13 patients)	
I	2 (15.4%)
II	4 (30.8%)
III	4 (30.8%)
IV	3 (23%)

Unless otherwise noted, data are numbers of patients, with percentages in parentheses

### Clinical and instrumental evaluation

All enrolled patients underwent a complete clinical and laboratory evaluation with lower and upper GI endoscopy. In 13/21 patients (61.9%), GI-aGVHD was confirmed at biopsy. Of these, 2/13 patients (15.4%) had grade I; 4/11 (30.8%), grade II; 4/11 (30.8%), grade III; and 3/11 (23%), grade IV disease, according to clinical grading.

In 8/21 patients, the diagnosis of GI-aGVHD was not confirmed at biopsy: two patients were found to have cytomegalovirus colitis, two were found to have *Clostridium Difficile* colitis, two were successfully treated for nonspecific infective colitis, and no definite diagnosis was established in two patients, who fully recovered at follow-up.

### Imaging evaluation

Among MRI findings considered valuable for the diagnosis, the inflammatory involvement of the small bowel was the most important indicator of GI-aGVHD, being present in 92.3% of patients (12/12); 84.6% of patients had ileal or ileocolonic intestinal involvement, whereas only 7.7% showed exclusive colonic involvement. The mean number of involved intestinal segments in GVHD patients was 7.15 (range 3–12). The most frequently affected intestinal segments were the distal, the middle, and the proximal ileum (Table 4).

**Table 4:** Different intestinal sites involved in the two populations: with GVHD and without GVHD

Involved segment	No. of GVHD patients	No. of non-GVHD patients
Stomach	1/13 (7.7%)	0/8 (0%)
Duodenum	8/13 (61.5%)	1/8 (12.5%)
Jejunum	9/13 (69.2%)	1/8 (12.5%)
Proximal ileum	11/13 (84.6%)	1/8 (12.5%)
Middle ileum	11/13 (84.6%)	1/8 (12.5%)
Distal ileum	11/13 (84.6%)	2/8 (25%)
Cecum	7/13 (53.8%)	6/8 (75%)
Ascending colon	8/13 (61.5%)	6/8 (75%)
Transverse colon	6/13 (46.2%)	5/8 (62.5%)
Descending colon	8/13 (61.5%)	7/8 (87.5%)
Sigmoid colon	6/13 (46.2%)	7/8 (87.5%)
Rectum	6/13 (46.2%)	8/8 (100%)

**Fig. 2:**
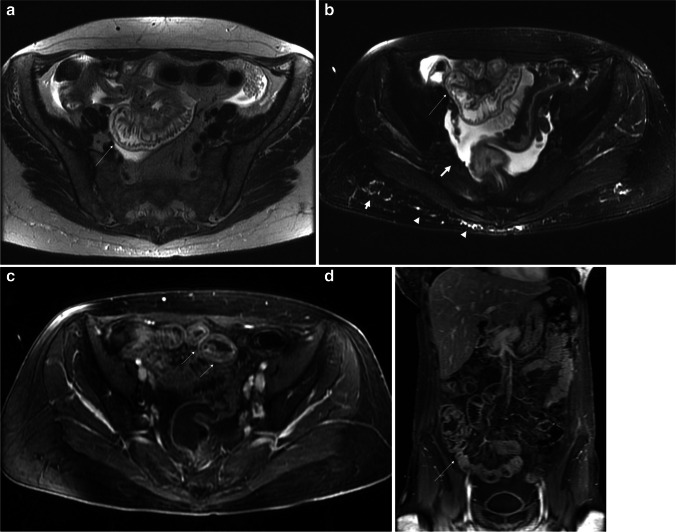
Acute intestinal GVHD in a 40-year-old woman, Glucksberg grade I (low clinical grade). The calculated MRI severity score is 12, considered as a moderate-grade disease (low mortality risk). Only the ileum is involved. **a** (T2-weighted axial image) and **b** (T2-weighted fat-suppressed axial image) show mild thickening with *edema and stratification* of the bowel wall (thin arrows); in addition, in the fat-suppressed image, a slight *ascites* and mild *edema of the declivous subcutaneous* (arrowheads) can be observed. **c** (T1-weighted gadolinium-enhanced axial image) and **d** (T1-weighted gadolinium-enhanced coronal image) show post-contrast enhancement and stratification at the level of the affected bowel (thin arrows)

Additional radiological features valuable for the diagnosis were: stratification of the affected bowel wall in T2-weighted images (*p* = 0.014), post-gadolinium wall stratification in T1-weighted images (*p* = 0.004), ascites (*p* = 0.04), edema of the retroperitoneal fat tissue (*p* = 0.001), and edema of the declivous tissues (*p* < 0.001) (Fig. 2).

Reading radiologists, after the unblinding to clinical results and in agreement with the statisticians, agreed on consensus that the most significant imaging findings for the diagnosis of GVHD were the following six:

1. Small-bowel involvement (calculated as the sum of the involved segments);
2. Intestinal wall stratification on T2-weighted images (suggestive of submucosal edema);
3. Post-contrast wall stratification on T1-weighted images, acquired in the arterial phase (suggestive of intestinal hypervascularity and edema);
4. Peritoneal fluid (related to peritoneal inflammation);
5. Edema of the retroperitoneal fat tissue (related to peritoneal inflammation);
6. Edema of the declivous muscular and subcutaneous tissues (related to severe diarrhea).

**Fig. 3:**
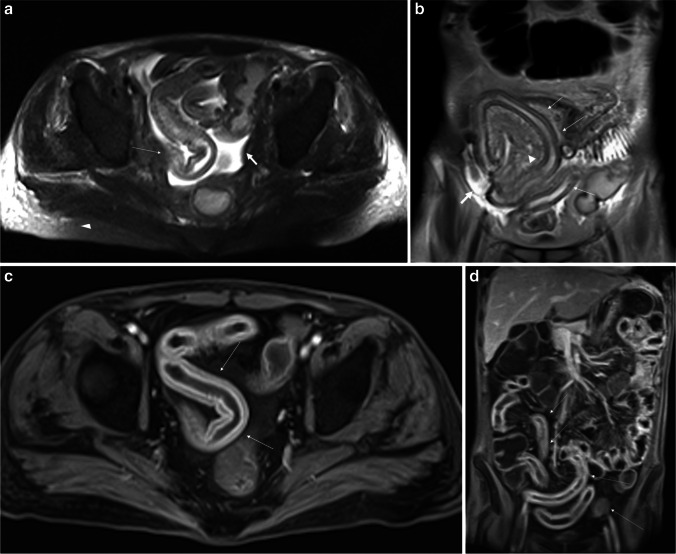
Acute intestinal GVHD in 36-year-old male, Glucksberg grade II (moderate clinical grade). The MRI severity score is 20 and considered as moderate-to-severe disease. The entire ileum is involved. **a** (T2-weighted fat-suppressed axial image) and **b** (T2-weighted coronal image) show diffuse continuous *edema with stratification of the bowel wall (thin arrows)*, mild ascites (thick arrows), and edema of the mesenteric fat tissue and of declivous tissues (arrowheads). **c** (T1-weighted gadolinium-enhanced axial image) and **d** (T1-weighted gadolinium-enhanced coronal image) show marked wall contrast enhancement and wall stratification (thin arrows). All the pictures clearly show the diffuse stiffness* and *continuous involvement* of the bowel loops (thin arrows). *Intestinal stiffness: intended as a continuous diffuse and circumferential intestinal wall edema (continuous wall thickening) which causes a serpiginous and rigid appearance of the involved bowel, showing smooth rather than sharp angles

**Fig. 4:**
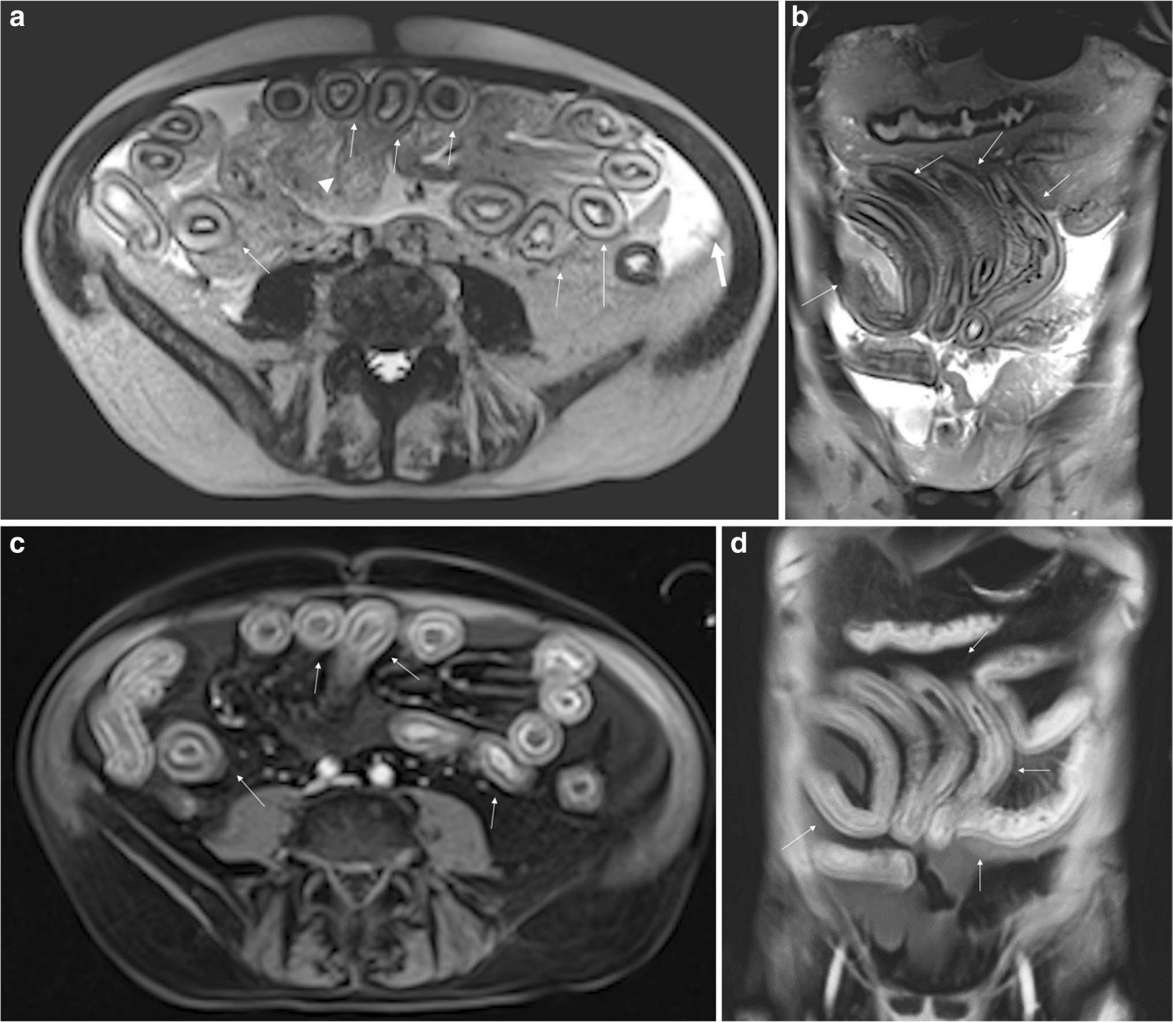
Acute intestinal GVHD in a 56-year-old man, Glucksberg grade IV (severe disease, high mortality risk). The MRI severity score is very high (24) and classified as “severe” disease (high mortality risk). The entire small intestine is involved. The patient died a few days later. **a** (T2-weighted axial image) and **b** (T2-weighted coronal image) show diffuse concentric *wall thickening and marked stratification of the intestinal wall* and *wall edema (thin arrows)*, *ascites* (thick arrows), and edema of retroperitoneal tissues and mesenteric fatty tissue (arrowhead). **c** (T1-weighted gadolinium-enhanced axial image) and **d** (T1-weighted gadolinium-enhanced coronal image) show severe *wall thickening with marked wall contrast enhancement and stratification* (thin arrows). The coronal plane clearly shows the stiffness* of the involved intestinal loops. *Intestinal stiffness: intended as a continuous diffuse and circumferential intestinal wall edema (continuous wall thickening) which causes a serpiginous and rigid appearance of the involved bowel, showing smooth rather than sharp angles

**Fig. 5:**
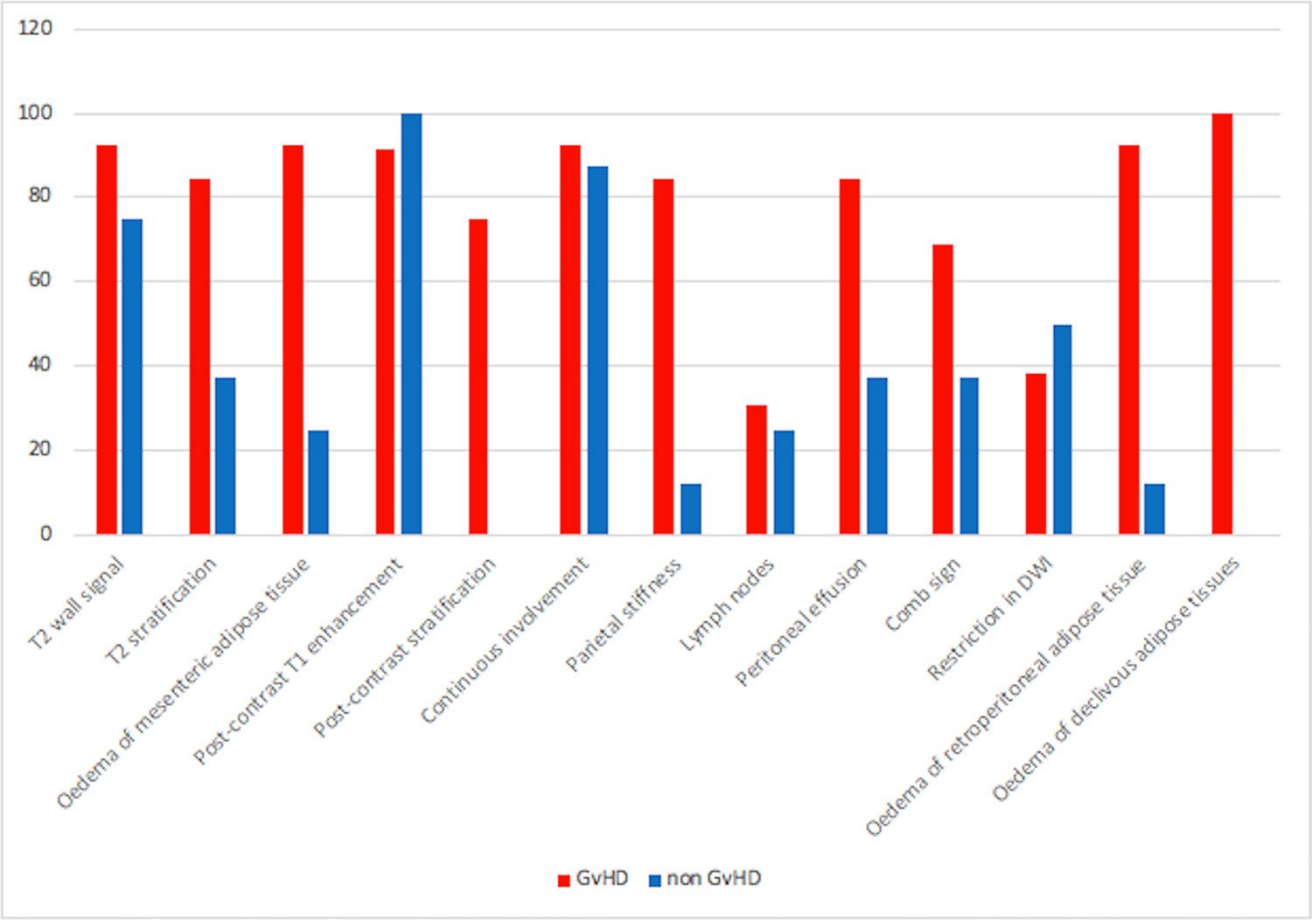
The graph shows the presence or absence of 13/15 MR signs in the two groups of patients with and without GVHD. Two/15 signs are not included in the graph: the number of segments involved and maximum thickness, because they represent continuous variables. Note that T2 and post-contrast wall stratification, ascites, retroperitoneal tissue edema, and declivous tissue edema are statistically different in the two groups as well as mesenteric edema and bowel stiffness (see Table 2)

Details are reported in Table 2 and shown in Figs. 2, 3, 4, and 5. The sum of these parameters (MRI diagnostic score) ranged between 0 and 14. According to Youden’s *J* statistic, patients with a score greater than or equal to 7 were considered positive for GI-aGVHD.

By using these six specific parameters, a GI-aGVHD MRI diagnostic score was developed, which correctly diagnosed the disease in 11/13 cases (84.6%). Overall, it showed 84.6% sensitivity (95% CI 69–100) and 100% specificity (95% CI 75–100) in identifying GI-aGVHD (AUC = 0.962, 95% CI 0.891–1), with excellent discriminatory ability and statistical significance.

Two additional parameters were significantly related to GI-aGVHD: the edema of the mesenteric fat (*p* = 0.006) and the intestinal wall stiffness (*p* = 0.005), intended as a continuous diffuse and circumferential intestinal wall edema which causes a serpiginous and rigid appearance of the involved bowel, showing smooth rather than sharp angles (Table 2, Figs. 3 and 4).

Comparing the two patients’ populations (with/without GI-aGVHD), it emerges that those with aGVHD had multiple continuously involved intestinal segments, mild-to-moderate circumferential wall thickness (mean of 5.23, SD 1.39), and wall stratification both on T2-weighted and T1-weighted post-gadolinium images; conversely, patients without GI-aGVHD had partial or entire colonic involvement with moderate-to-severe wall thickening (mean 7.88, SD 4.25) and mild or absent wall stratification; among these patients, 75% showed only colonic inflammation, while 25% had ileocolonic involvement (Table 2 and Fig. 5).

For scoring purposes, all the 15 parameters were considered relevant and summed to obtain a severity score (MRI severity score for GI-aGVHD) ranging from 0 to 27.

According to Youden’s *J* statistic, the optimal MRI severity score threshold for high clinical grade (Glucksberg III–IV) was 21/27, which showed 42.9% sensitivity (95% CI 0.14–1) and 83.3% specificity (95% CI 0.17–1) (AUC 0.595, 95% CI 0.256–0.934), thus not very sensitive for detecting high-clinical-grade GI-aGVHD. However, the highest (21/27) MRI severity score threshold for GI-aGVHD showed 100% sensitivity (95% CI 100; 100) and 90% specificity (95% CI 70;100) for 1-month related mortality (AUC = 0.933, 95% CI 0.787–1), superior to the clinical score. In fact, the highest MRI severity score was associated with a mortality of 75% whereas the highest clinical severity score was associated with a mortality of 42.8%. Three of the four GI-aGVHD patients with the highest MRI severity score (> 21) died because of GI-aGVHD (Fig. 4), whereas one survived despite very severe clinical GI-aGVHD. On the other hand, 3/7 GI-aGVHD patients with the highest clinical score (grade III–IV Glucksberg) died, whereas 4/7 survived.

### Reproducibility

Inter-reader concordance was satisfactory. The criteria to identify the six main MRI parameters are described in Table 2. The values of Fleiss’s kappa and intraclass correlation coefficients reported in Table 5 highlight the good agreement between the readers in the assessment of disease with MRI.

**Table 5:** Inter-observer reliability for MRI localization of diseased intestinal segments and for each of the 17 MRI parameters measured with Fleiss’ kappa (in the case of categorical and ordinal variables) and with intraclass correlation coefficient (for continuous variables)

	Inter-rater reliability measure	95% CI	*p* value
Intestinal segments			
Stomach	0*	(− 0.05, 0.018)	0.8981
Duodenum	0.6429*	(0.365, 0.921)	< 0.001
Jejunum	0.8727*	(0.69, 1)	< 0.001
Proximal ileum	1*	(1, 1)	< 0.001
Middle ileum	1*	(1, 1)	< 0.001
Distal ileum	1*	(1, 1)	< 0.001
Cecum	0.8603*	(0.665, 1)	< 0.001
Ascending colon	0.7905*	(0.549, 1)	< 0.001
Transverse colon	0.8083*	(0.59, 1)	< 0.001
Descending colon	0.9267*	(0.776, 1)	< 0.001
Sigmoid colon	0.7308*	(0.473, 0.988)	< 0.001
Rectum	0.7946*	(0.563, 1)	< 0.001
MRI features analyzed			
Number of involved segments	0.8851**	(0.76, 0.95)	< 0.001
Grade of involved segments	0.5932*	(0.354, 0.832)	< 0.001
Maximum wall thickness	0.9775**	(0.95, 0.99)	< 0.001
Grade of wall thickness	0.6909*	(0.464, 0.918)	< 0.001
Wall T2w signal	0.6582*	(0.393, 0.924)	< 0.001
T2w edema of the mesenteric adipose tissue	0.6156*	(0.39, 0.841)	< 0.001
Post-contrast T1 wall enhancement	0.726*	(0.452, 0.986)	< 0.001
Post-contrast parietal stratification	0.8667*	(0.654, 1)	< 0.001
Continuous intestinal involvement	0.7331*	(0.141, 1)	< 0.001
Parietal stiffness	1*	(1, 1)	< 0.001
DWI	0.5788*	(0.232, 0.645)	< 0.001
Increased number and or size of mesenteric lymph nodes	0.4983*	(0.192, 0.805)	< 0.001
Comb sign	0.619*	(0.338, 0.9)	< 0.001
Peritoneal effusion	0.7581*	(0.552, 0.964)	< 0.001
T2w wall stratification	0.8551*	(0.691, 1)	< 0.001
Edema of retroperitoneal adipose tissue	0.9504*	(0.848, 1)	< 0.001
Edema of declivous tissues (muscle and subcutaneous tissues)	0.8518*	(0.686, 1)	< 0.001

*Fleiss’ kappa

**Intraclass correlation coefficient (ICC)

## Discussion

In this study, we developed two MRI scores for GI-aGVHD: one for diagnosis, which showed 84.6% sensitivity and 100% specificity, and the other for staging of disease severity, which showed a higher prognostic power than the more commonly used clinical score. Both scores could play a crucial role in the management of the disease.

 The first, named *MRI diagnostic score*, is rather simple, being based on the six most significant parameters for GI-aGVHD (small-bowel inflammatory involvement, parietal stratification on T2-weighted sequences, post-contrast parietal stratification, ascites, declivous tissue edema, and retroperitoneal edema).

The second, named *MRI severity score*, is more extensive, being based on fifteen disease-related MRI signs. This score did not show a satisfactory statistical correlation with the clinical score system (the Glucksberg score), but rather showed a good prognostic value, with a correlation with disease mortality higher than that of the clinical score. These results, although obtained in a very small patient population, indicate that MRI could predict clinical outcome more accurately than the clinical severity score itself. Indeed, it is reasonable to hypothesize that quantification of 15 different disease features evident on MRI images may predict disease severity more accurately than the daily volume of diarrhea, which is the main marker of the clinical severity score. Although based on a broad scale ranging from 1 to 27 points, the MRI severity score was stratified into two main groups only: patients at high risk of death from intestinal GVHD (score above 21 points) and low-risk patients. Further stratification of the lower-risk group will likely be available in the future on larger series.

Thus far, only few studies have investigated the role of imaging in the diagnosis of intestinal GVHD, most of them performed with CT, very few with MRI [19–28]. Currently, however, MRI plays a primary role in the diagnosis of intestinal inflammation, superior to CT. In IBD, MRI is currently considered analogous or even superior to endoscopy itself in assessing the effects of medical treatment [33, 41]. Given the similarity between GI-aGVHD and IBD [35, 36], it is legitimate to assume that MRI may play an important role also in GI-aGVHD.

To the best of our knowledge, only two previous studies have investigated the diagnostic accuracy of MRI in acute GI-GVHD, based on patient populations similar in size to ours [27, 28]. Both reported similar results to ours regarding the main diagnostic features of the disease: wall thickening, wall enhancement, and mural stratification in the small and large bowel. In the first study [27], which focused only on acute patients (9 positive and 11 negative), Budjan et al also reported, as a main sign of GI-aGVHD, a continuous inflammatory bowel involvement, in full agreement with our results.

In the second study [28], Derlin et al reported an overall 65.9% MRI accuracy, lower than ours, with 81.5% sensitivity and 35.7% specificity, likely due to the lower homogeneity of the population in analysis, which included both acute (9) and chronic (18) GI-GVHD patients. Interestingly, in agreement with our study, the authors reported a statistical correlation between the number of involved segments (*r*s = 0.54, *p* = 0.009) and the clinical grading, suggesting a potential clinical role of MRI, although they did not propose a final severity score [28].

Compared with previous MRI studies [26–28], ours is innovative for several reasons. It explores new morphological and activity markers for the diagnosis of GI-aGVHD, such as bowel wall stiffness, retroperitoneal adipose tissue edema, declivous tissue edema, and mesenteric adipose tissue edema, features observed in the majority of our patients and never investigated before. Furthermore, although both previous studies suggested an important diagnostic value of MRI in the evaluation of GI-aGVHD severity, none of them suggested a scoring system.

Our study has one main limitation: it was conducted on a relatively small sample of patients due to the extreme rarity of the disease, which involves only a small percentage of the most severe hematologic patients. For the same reasons, the study is retrospective, as it is difficult to design a prospective study on this specific and rare patient population. The two previously published studies on MRI in acute GI-GVHD, however, were based on similar sample sizes. We expect larger multicenter and prospective studies with greater statistical power to confirm these results.

In conclusion, MRI has proved to be an effective diagnostic tool for diagnosing and scoring GI-aGVHD, with a high prognostic value. In the coming years, if these results are confirmed, MRI could partly replace endoscopy, thus becoming the primary diagnostic tool for GI-aGVHD, being more comprehensive, less invasive, and more easily repeatable after therapy.

## Abbreviations

aGVHD: Acute graft-versus-host disease
CI: Confidence interval
GI: Gastrointestinal
GI-aGVHD: Gastrointestinal acute graft-versus-host disease
GVHD: Graft-versus-host disease
HSCT: Hematopoietic stem cell transplantation
IBD: Inflammatory bowel disease
IRR: Inter-rater reliability
IV: Intravenous
SD: Standard deviation

## Declarations

### Guarantor

The scientific guarantor of this publication is Professor Francesca Maccioni.

### Statistics and biometry

Two of the authors have significant statistical expertise (Alberto Milanese and Anna Rita Vestri).

### Methodology

• retrospective

• diagnostic or prognostic study

• performed at one institution
